# Cultivation and energy efficient harvesting of microalgae using thermoreversible sol-gel transition

**DOI:** 10.1038/srep40725

**Published:** 2017-01-19

**Authors:** Bendy Estime, Dacheng Ren, Radhakrishna Sureshkumar

**Affiliations:** 1 Department of Biomedical and Chemical Engineering, Syracuse University, Syracuse, New York 13244, USA; 2 Department of Civil and Environmental Engineering, Syracuse University, Syracuse, New York 13244, USA; 3 Department of Biology, Syracuse University, Syracuse, New York 13244, USA; 4 Syracuse Biomaterials Institute, Syracuse University, Syracuse, New York 13244, USA; 5 Department of Physics, Syracuse University, Syracuse, New York 13244, USA

## Abstract

Microalgae represent a promising source of renewable biomass for the production of biofuels and valuable chemicals. However, energy efficient cultivation and harvesting technologies are necessary to improve economic viability. A Tris-Acetate-Phosphate-Pluronic (TAPP) medium that undergoes a thermoreversible sol-gel transition is developed to efficiently culture and harvest microalgae without affecting the productivity as compared to that in traditional culture in a well-mixed suspension. After seeding microalgae in the TAPP medium in a solution phase at 15 °C, the temperature is increased by 7 °C to induce gelation. Within the gel, microalgae are observed to grow in large clusters rather than as isolated cells. The settling velocity of the microalgal clusters is approximately ten times larger than that of individual cells cultured in typical solution media. Such clusters are easily harvested gravimetrically by decreasing the temperature to bring the medium to a solution phase.

The development of methods for high throughput cultivation and efficient harvesting of microalgae has, over the past decades, constituted an active field of research^1^,^2^. Despite major advances, there is still a need to optimize and increase productivity in microalgal cultivation systems in order to make microalgal biofuels production a more viable option^3^,^4^. It is also imperative to improve microalgal harvesting processes which currently account for about thirty percent of total production cost^5^.

Many cultivation methods have been proposed to improve microalgal biomass production. For instance, growth medium modifications with high salt and nutrient deprivation have been used to enhance accumulation of specific chemicals such as lipids and carbohydrates^6^,^7^. Furthermore, biofilm and biofouling of microalgae that are often portrayed as challenges for suspended culture have recently been explored as cultivation methods for large-scale microalgal biomass production^8^. Among many others, the large decrease in water consumption and the simplification of the harvesting process are considered as two major benefits of biofilm cultivation of microalgae^9^,^10^. As for suspended cultivation, constant mixing is usually necessary during the entire cultivation period and the current harvesting methods often involving centrifugation, pumping or electrophoresis techniques are largely energy intensive. The alternatives that have been proposed thus far are yet to resolve the energy consumption issue^11^.

Motivated by the need for energy efficient microalgal cultivation and harvesting technologies, we aimed at exploring a microalgal cultivation and harvesting strategy using the thermoreversible copolymer pluronic. Pluronic is an amphiphilic ABA type copolymer composed of both hydrophobic Polypropylene Oxide (PPO, B) block parts and hydrophilic Polyethylene Oxide (PEO, A) block parts known for its good biocompatibility and low toxicity^12^. The applications of this copolymer are highly diversified. For example, the copolymer pluronic F-127 is believed to be a good carrier for drug delivery and is therefore valuable in pharmaceutical formulations^13^. Pluronic has also largely been investigated for its potential in controlling biofouling^14^. Moreover, this copolymer is well known for its effectiveness in producing stable surface patterns and can be useful in long term single-cell culture^15^,^16^. Note that single-cell cultivation of microalgae has been proposed as a good method for preparing colonies of promising strains for large-scale cultivation^17^.

The temperature dependent sol-gel transition behavior of the copolymer pluronic along with the largely reported biocompatibility inspired its use in microalgal cultivation. An aqueous solution of pluronic would robustly undergo a phase transition to an elastic gel when heated above a gelation temperature T_g_. This gelation process is induced by a thermodynamic self-assembly of the copolymer molecules into an inter-connected micellar network and is reversible, i.e., the gel can be brought back into the solution phase by cooling it below T_g_^18^. Depending on the concentration of the pluronic polymer in the aqueous phase, T_g_ values range between 15 °C to 30 °C^19^,^20^. This intersects with the temperature range often used for microalgal cultivation.

Herein, a thermoreversible Tris-Acetate-Phosphate-Pluronic (TAPP) medium for energy efficient cultivation and harvesting of microalgae is presented. The thermorheological properties of the pluronic-based TAPP medium as well as the resulting pluronic-microalgae matrix after cultivation are systematically characterized. Further, cultivation experiments are performed using microalga *Chlamydomonas reinhardtii* and microalgal biomass production in the TAPP medium is assessed both qualitatively and quantitatively. Furthermore, a framework is proposed to efficiently harvest the microalgal biomass produced through relatively small variations of temperature (Fig. 1). Finally, the microalgal biomass harvesting parameters are characterized and the harvesting efficiency is quantified using the experimental results.

## Results

### Rheological characterization of the TAPP medium

In order to obtain a range of pluronic concentrations that can confer the suitable properties necessary for the proposed thermoreversible microalgal cultivation and harvesting system, TAPP media with different pluronic concentrations were prepared and were subjected to rheological testing. Specifically, the linear viscoelastic properties, namely the storage (G′) and loss (G′′) moduli were measured as a function of temperature T using a small amplitude oscillatory shear flow experiment. In the viscous solution phase, G′′ >> G′ The gel point T_g_ (or the critical micellation temperature, CMT) is defined as the temperature for which G′ = G′′^21^. For T > T_g_, G′ exceeds G′′ and reaches a plateau. Further, the zero-shear viscosity η_0_, i.e., viscosity of the samples under very small shear rates (near-equilibrium conditions), was also measured. Gelation is accompanied by a sharp increase in η_0_.

Typical results for G′, G′′ and η_0_ obtained from dynamic temperature ramp experiments are reported in Fig. 2. For low T, the moduli of pluronic-based media are relatively low. It is in fact known that, at low T, pluronic in water solution tends to adopt the form of a unimer^22^. Therefore, low entanglement between the chains would lead to low moduli. As the temperature was increased, the breakage of hydrogen bonds and aggregation of hydrophobic PPO blocks stimulated gelation and led to sharp increases in the moduli (by up to six orders of magnitude)^23^. The gel points T_g_ were found to be 20.1 °C, 21.8 °C and 23.9 °C respectively for the 22, 20 and 18 weight percent pluronic in TAPP media samples (Fig. 2a). The decrease in T_g_ with increasing concentration can be attributed to an increase in molecular entanglements with increasing concentration^24^. The viscosity profiles of the pluronic-based media exhibited a behavior similar to that of the moduli during the temperature ramp experiment where the viscosity increases significantly for T > T_g_ (Fig. 2b). This is likely due to the fact that with an increase in temperature the polymer chains dehydrate and begin to cross-link leading to a closely packed polymeric network^25^,^26^. Accordingly, the mesh size of the network decreases with increasing temperature and increasing pluronic concentrations with values ranging from tens to thousands of nanometers^27^.

### Microalgal biomass production in the TAPP medium

The wild type microalga *Chlamydomonas reinhardtii* CC-124 was allowed to grow in the TAPP media with 3 different pluronic concentrations (18, 20 and 22 weight percent) and a traditional well-mixed TAP medium culture, used as a control. Therefore, at the operating temperature (22 °C), the 18% TAPP medium would still be in early micellation stage (i.e., a viscous liquid), the 20% TAPP medium would be close to T_g_ (i.e., a soft gel) and the 22% TAPP sample in an elastic solid-like state. The initial microalgal biomass concentration for all the samples was set to 0.1 g l^−1^. Microalgal growth was assessed through optical density (OD_675_) analyses and gravimetric measurements. Microalgal biomass concentrations, after a seven day cultivation period, were found to be 3.1 ± 0.2 g l^−1^, 2.9 ± 0.3 g l^−1^, 2.8 ± 0.4 g l^−1^ and 2.9 ± 0.4 g l^−1^, respectively for the well-mixed TAP medium control, the 18% TAPP, the 20% TAPP and the 22% TAPP samples (P > 0.05) (Fig. 3a). The content in lipid and carbohydrate of the microalgal biomass generated did not vary significantly (P > 0.05) under these four conditions (Fig. 3b). These results indicate that microalgae grow well in the TAPP medium with no statistically significant variations in the composition of the generated biomass unlike those commonly observed in many other experiments with growth medium modification^6^. Microalgal growth was also tested in a pluronic-based medium with an inorganic carbon source. Towards this end, microalgae were grown in a minimum medium enriched with 20 mM of sodium bicarbonate (NaHCO_3_) and 22% (weight) pluronic. Note that this medium had the same composition as the TAPP medium except that in this case sodium bicarbonate was used as the carbon source in lieu of acetic acid. Under the same culture conditions, the pluronic-based medium with inorganic carbon source led to a microalgal biomass production of 1.7 ± 0.3 g l^−1^ whereas a control minimum medium enriched with 20 mM of sodium bicarbonate not containing pluronic led to a microalgal biomass production of 1.9 ± 0.2 g l^−1^ (P > 0.05). This proves that pluronic-based media with organic as well as inorganic carbon sources are conducive for the growth of microalgae.

The shape and size of microalgal cells confined in the TAPP media were assessed through microscopic analyses. While there was no significant change (P > 0.05) in the shape of the cells, their size decreased significantly (P < 0.05) when growing in the TAPP medium. Average cell diameters were found to be 8.1 ± 0.6 μm, 6.9 ± 0.5 μm, 6.7 ± 0.4 μm and 6.3 ± 0.5 μm, respectively for growth in the well-mixed TAP medium control, the 18% TAPP, the 20% TAPP and the 22% TAPP samples (Fig. 3c). This decrease in cell size could be attributed to confinement of the microalgal cells by the surrounding polymeric network. In fact, at the operating conditions, the three TAPP samples were at different micellation stages and their elastic moduli and mesh sizes exhibited significant differences, which corroborate the observed variation in cell size^27^. Nonetheless, the decrease in cell size does not undermine the fact that the thermoreversible polymer pluronic F-127 can be used in microalgal cultivation since the biomass productivity and composition did not exhibit any significant differences from the control.

### Influence of microalgal proliferation on the thermorheological behavior of the TAPP medium

The effects of proliferation of microalgal cells on the thermorheological properties of the TAPP medium were also assessed since large variations on such properties might impact its applicability in microalgal cultivation and harvesting. For this reason, rheological measurements were made on the microalgae-TAPP matrix resulting from the seven day cultivation period. It was observed that, with all three pluronic concentrations (22, 20 and 18 weight percent pluronic), there was a slight decrease in T_g_ in presence of microalgal cells. Specifically, T_g_ decreased from 20.1 °C to 19.2 °C, 21.8 °C to 20.9 °C and 23.7 °C to 22.8 °C respectively for the 18% TAPP, the 20% TAPP and the 22% TAPP samples (Fig. 4a,b). This is likely due to the hydrophobic interactions between microalgal cell walls and the hydrophobic PPO blocks of the pluronic molecules resulting in an acceleration of the micellation process^28^. Nonetheless the decrease in the T_g_ is relatively small and does not affect the microalgal cultivation and harvesting.

### Harvesting and recultivation of microalgae using thermoreversible sol-gel transition

**Figure 1:**
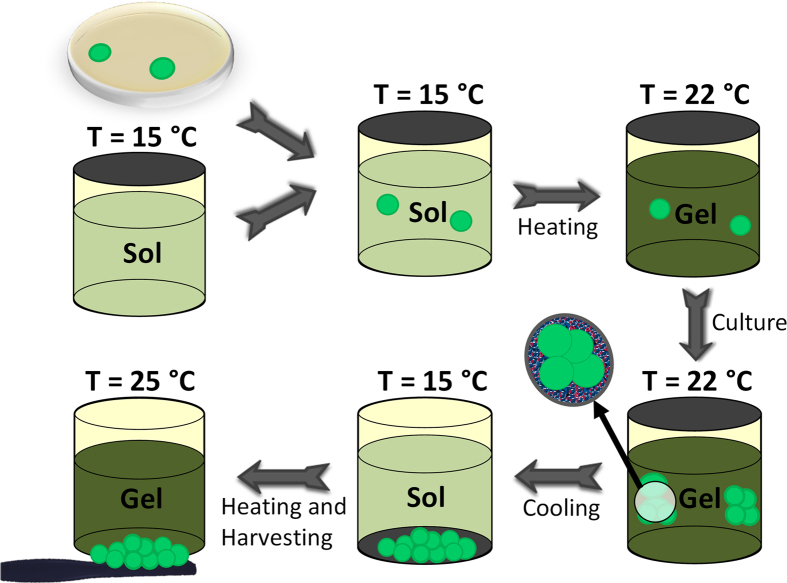
Schematic of microalgal cultivation and harvesting process using thermoreversible sol-gel transition. Microalgal cells are seeded in the TAPP medium in solution phase at 15 °C. Then, the temperature is raised at 22 °C for gelation of the medium and entrapped microalgal cultivation. After the cultivation period, the temperature is decreased to 15 °C allowing microalgal clusters to gravimetrically settle at the bottom. The temperature is finally raised to 25 °C and settled microalgal clusters are scraped off the TAPP surface.

**Figure 2:**
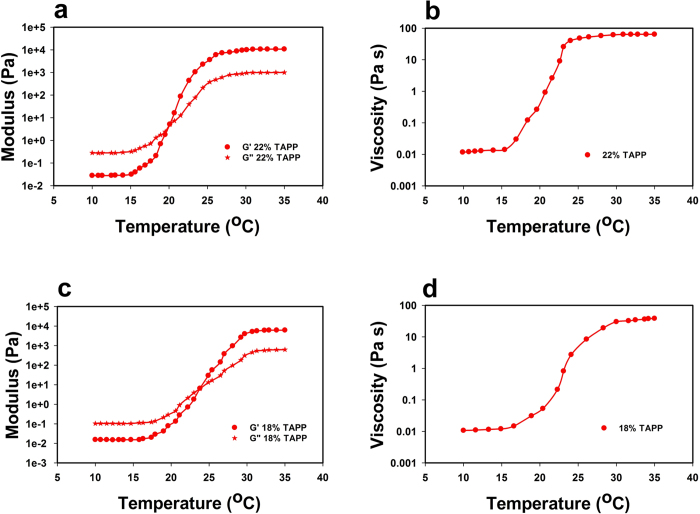
Thermorheological properties of the TAPP medium. (**a**) Storage modulus (circle) and loss modulus (star) of the 22% pluronic in TAPP sample as a function of temperature, (**b**) viscosity profile of the 22% pluronic in TAPP sample as a function of temperature, (**c**) storage modulus (circle) and loss modulus (star) of the 18% pluronic in TAPP sample as a function of temperature and (**d**) viscosity profile of the 18% pluronic in TAPP sample as a function of temperature.

**Figure 3:**
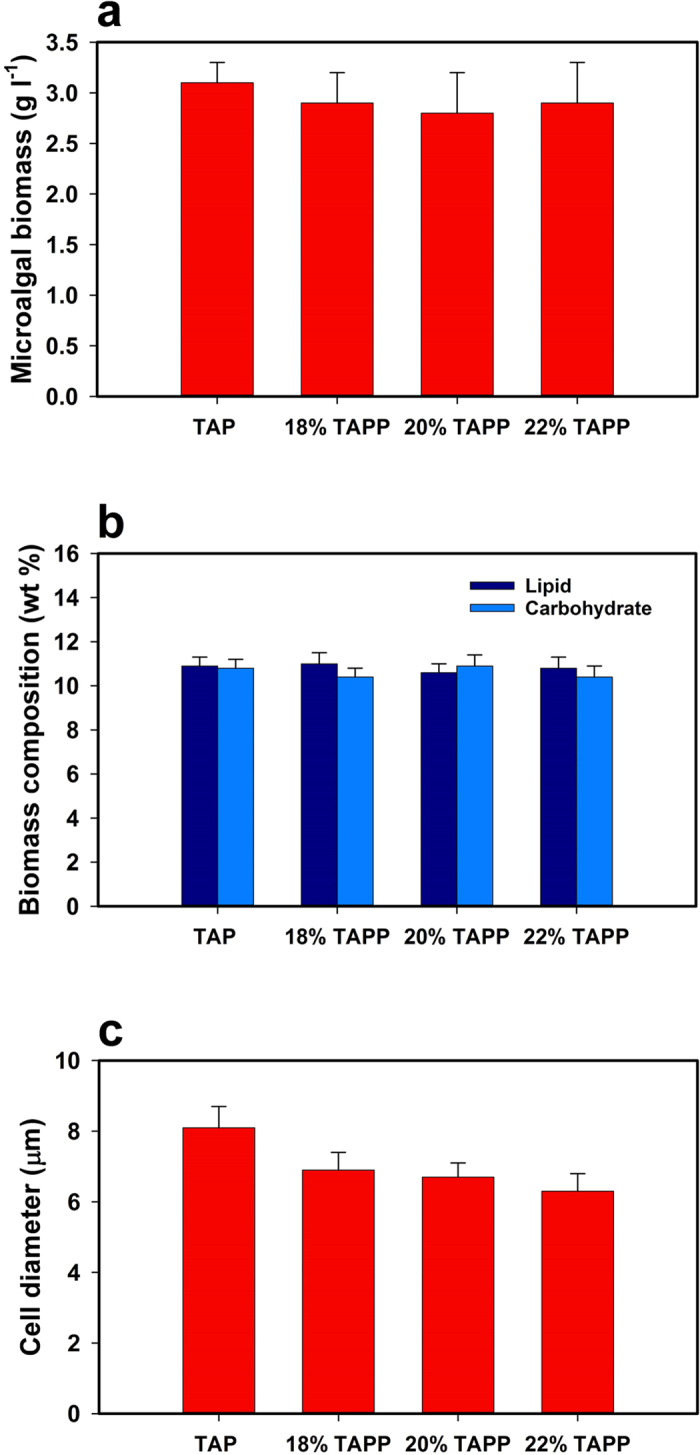
Microalgal biomass production in the TAPP medium. (**a**) Microalgal biomass generation (g l^−1^), (**b**) weight percentage of lipid and carbohydrate in microalgal biomass and (**c**) average diameter of microalgal cells growing in the well-mixed TAP control, the 18% TAPP, the 20% TAPP and the 22% TAPP. Bars represent means of 30 measurements and error bars are one S.D.

**Figure 4:**
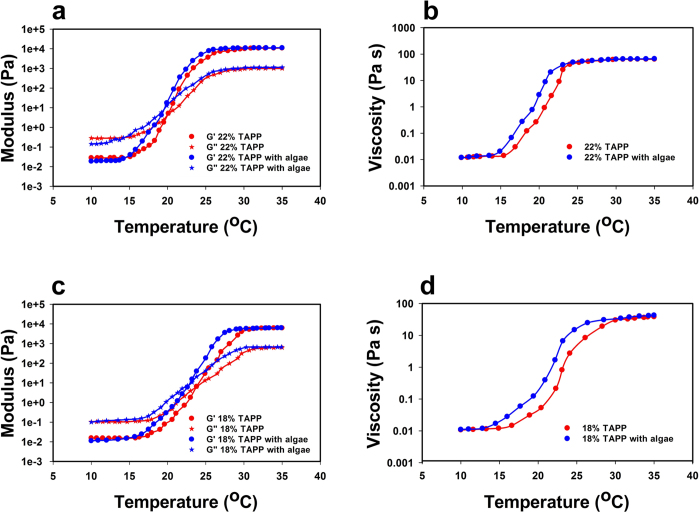
Effects of microalgal proliferation on the thermorheological behavior of the TAPP medium. (**a**) Storage modulus (circle) and loss modulus (star) of the 22% pluronic in TAPP sample, with (blue) and without (red) microalgae, as a function of temperature, (**b**) viscosity profile of the 22% pluronic in TAPP sample, with (blue) and without (red) microalgae, as a function of temperature, (**c**) storage modulus (circle) and loss modulus (star) of the 18% pluronic in TAPP sample, with (blue) and without (red) microalgae, as a function of temperature, (**d**) viscosity profile of the 18% pluronic in TAPP sample, with (blue) and without (red) microalgae, as a function of temperature.

One of the major advantages of the TAPP medium is the potential for a simple and efficient microalgal harvesting process resulting from the robust thermoreversible sol-gel transition. Specifically, the fact that this transition is completely reversible allows one to control confinement and/or settlement of microalgae through relatively small changes in the system temperature. As illustrated in the schematic (Fig. 1), the cultivation and harvesting experiment involved seeding microalgae at a temperature where the TAPP medium is still in a solution phase and increasing the temperature beyond (or around) T_g_ to allow microalgal cells to grow in a confined environment. After a fixed cultivation period, the temperature was decreased below T_g_ and microalgae settled to the bottom of the cultivation chamber, thereby allowing for a *gravimetric separation of* the microalgal cells from the culture medium. Subsequently, the temperature was increased to jellify the supernatant and harvesting of microalgae simply involved scraping microalgal flocs off the TAPP surface.

The distribution of microalgal cells within the TAPP medium was an important parameter for the proposed harvesting technique. We hypothesized that under the selected cultivation conditions microalgal cells would be distributed in clusters due to confinement effects as opposed to randomly distributed cells as it is the case in a well-mixed TAP suspension. The morphology of the clusters would impact the velocity with which they settle during the gravimetric separation stage of the process with spherically shaped clusters settling faster than anisotropic ones^29^. Furthermore, there is a direct correlation between the size of the clusters and the settling velocity, both according to Stokes’ law or the empirical formulae often used to determine settling velocity beyond the Stokes regime^30^.

**Figure 5:**
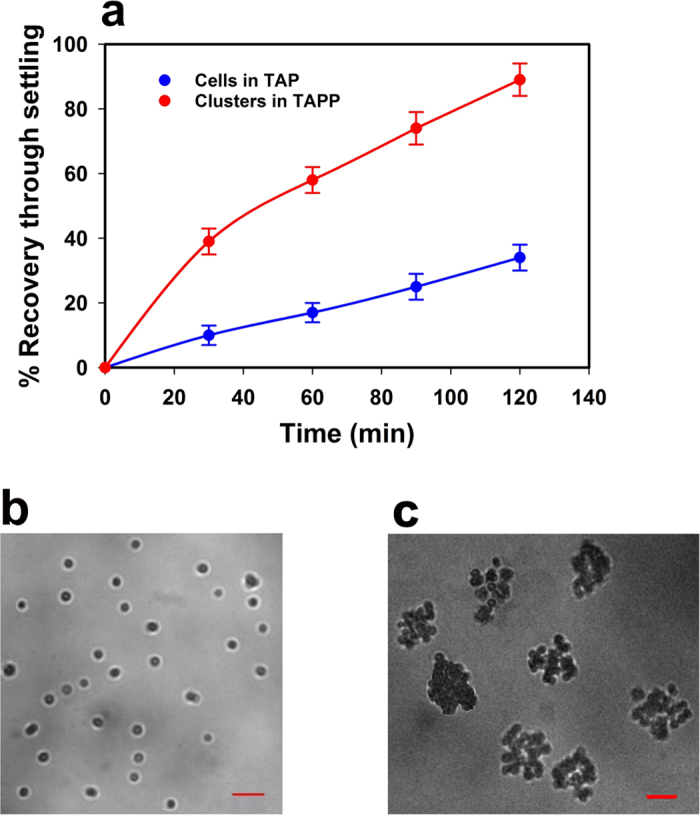
Characterization of microalgal settling. (**a**) Percentage of microalgal biomass recovery through settling determined through results from optical density measurements at regular intervals. Data points represent means of 30 measurements and error bars are one S.D. (**b**) Image of microalgal cells in the well-mixed TAP medium and (**c**) image of microalgal cell clusters in the TAPP medium. Scale bars are 50 μm.

The distribution of microalgal cells was assessed through random selection of images captured with an Axio Imager M1 microscope (Carl Zeiss Inc., Berlin, Germany) on each batch of microalgal culture. The images were then processed and the shape and size of cells and clusters were characterized with a Zen pro software (Carl Zeiss Inc., Berlin, Germany). As hypothesized, microalgal cells from the TAPP system were observed to be organized into clusters (Fig. 5c) whereas those from the control (TAP medium) were visualized as randomly dispersed cells (Fig. 5b). The average form factor of the microalgal clusters was found to be 0.98 ± 0.02 μm which suggests nearly-spherical clusters and justifies the use of Stokes’ law to predict the settling velocity. The average equivalent diameter of the clusters was found to be 78 ± 9 μm compared to an 8.1 ± 0.6 μm average diameter for isolated cells in the well-mixed TAP medium. Considering the viscosity measured during the rheological characterization, the settling velocity was calculated according to Stokes’ law and averaged a value of 2.6 ± 6 m day^−1^ which is about ten times greater than the estimated settling velocity of isolated microalgal cells in TAP medium (0.27 ± 0.03 m day^−1^). To verify the settling rate experimentally, microalgae were allowed to settle at 15 °C in tubes with 10 cm working height after the seven day cultivation period. The optical density (OD_675_) of the TAPP broth was measured at regular intervals and the variation in microalgal biomass concentration was compared against a TAP broth used as control. The optical density measurements during the settling assay were used to compute the percentage recovery through settling over time. It was found that over a 2-hour period, 89 ± 5% of microalgal clusters in the TAPP broth were recovered through settling compared to 34 ± 3% for isolated cells in the TAP broth for the 10 cm working height (Fig. 5a). It is clear that these experimental results exhibited slower biomass recovery compared to the predictions using Stokes’ law. Similar deviations of experimental data from predicted settling rate are often reported and may be due to the large heterogeneity of cluster and cell sizes^31^ as well as due to hydrodynamic interactions among the clusters that modify the underlying flow field in the settling column. Nonetheless our experiments offer clear corroboration for a higher settling velocity of microalgal clusters in the TAPP medium compared to that of dispersed microalgal cells in the TAP medium.

To evaluate the harvesting efficiency through scraping of microalgae off the surface of the jellified TAPP system, two-capped containers may be used or one-capped containers may be flipped upside down before the decrease in temperature for clusters settling. The percentage of microalgae harvested was assessed through gravimetric measurements on the harvested biomass and also through optical density measurements on the remaining broth. The harvesting efficiency was then computed as the percent difference between microalgal concentrations of the broths prior and after harvesting. It was found that 89 ± 2%, 88 ± 3% and 86 ± 2% of microalgae were harvested respectively for the 22% TAPP, the 20% TAPP and the 18% TAPP samples.

Another advantage of the TAPP medium is the potential for recycling and reuse of the medium for recultivation of microalgae after the harvesting process. The thermoreversible sol-gel transition properties of the TAPP medium are not altered after cultivation and harvesting of microalgae. Therefore, recultivation of microalgae in the recycled TAPP medium simply requires a replenishment of nutrients based on the nutrient uptake in the preceding microalgal culture^32^,^33^,^34^. This was confirmed by recycling and reusing the TAPP medium for the recultivation of microalgae in a three cultivation cycles experiment. After each cultivation cycle, the microalgal biomass was quantified and harvested and the TAPP growth medium was recycled and reused for another cultivation. Starting with the same initial biomass concentration in each microalgal cultivation cycle, final microalgal biomass concentrations were found to be 2.8 ± 0.2 g l^−1^, 2.3 ± 0.3 g l^−1^ and 2.5 ± 0.3 g l^−1^ respectively for the first, second and third cultivation cycles.

## Discussion

In summary, a Tris-Acetate-Phosphate-Pluronic medium with thermoreversible sol-gel transition properties was developed for energy efficient cultivation and harvesting of microalgae. The thermorheological properties of the medium and the effects of microalgal proliferation on such properties were experimentally characterized to offer a framework for designing of robust microalgal cultivation and harvesting systems using the thermoreversible copolymer pluronic. Microalga *Chlamydomonas reinhardtii* was successfully cultivated in the TAPP medium and led to production of microalgal biomass with similar productivity, lipid and carbohydrate composition to that obtained from cultivation in a traditional well-mixed TAP suspension medium. In fact, starting with a same initial biomass concentration of 0.1 g l^−1^, microalgal biomass concentrations of 3.1 ± 0.2 g l^−1^, 2.9 ± 0.3 g l^−1^, 2.8 ± 0.4 g l^−1^ and 2.9 ± 0.4 g l^−1^, were obtained respectively with the well-mixed TAP medium control, the 18% TAPP, the 20% TAPP and the 22% TAPP samples after a seven day cultivation period (P > 0.05). The harvesting process of the microalgal biomass produced was highly simplified with the TAPP system. In fact, confinement of microalgal cells in the pluronic matrix led to a near-spherical cluster distribution that could increase their settling velocity by a factor of ten as compared to that of individual cells in well-mixed TAP medium. This allowed for an *energy efficient gravimetric separation* of the biomass from the culture medium. Through thermal cycles with relatively small variations in temperature within 7 °C, microalgal clusters were allowed to settle and harvesting of microalgae simply involved scraping the microalgal clusters off the surface of the jellified TAPP medium. These findings confirm that microalgae can be efficiently cultured in the TAPP system which does not require constant mixing like in typical solution broths. Moreover, this new process is capable of efficiently harvesting about ninety percent of the microalgal biomass produced with little energy input and is biocompatible. All of the above cultivation and harvesting features were also successfully verified with a pluronic-based medium with an inorganic carbon source to demonstrate that such thermoreversible media can be used for photoheterotrophic as well as photoautotrophic cultivation of microalgae. It is envisioned that thermoreversible TAPP-like systems could find further applications in microalgal biomass production for biofouling control and single-cell cultivation^14^,^15^,^16^. Future studies to continue this work may include potential functionalization of the copolymer in order to minimize the concentration necessary to confer the required thermorheological properties. Other studies may also be undertaken to elucidate the interactions of different microalgal species and strains with the pluronic matrix and to further characterize the microalgal clusters obtained through confinement in the TAPP matrix.

## Methods

### TAPP medium preparation and culture conditions

Pluronic F-127 was obtained from BASF (Ludwigshafen, Germany) and was used without further purification. The chemical composition for this pluronic type is PEO100PPO65PEO100 and the total molecular weight is 12600 g mol^−1^. PEO and PPO ratio is approximately 2:1 by weight. The pluronic-based growth medium was prepared in a way that maintains a concentration of nutrients similar to the traditional Tris-Acetate-Phosphate (TAP) medium with the addition of pluronic (TAPP medium) that confers the thermoreversible sol-gel transition properties. The concentrations of chemicals in the TAPP medium were therefore as follows: Tris (19.98 mM), NH_4_Cl (7.011 mM), MgSO_4_.7H_2_O (0.406 mM), CaCl2.2H2O (0.34 mM), K_2_HPO_4_.3H_2_O (0.47 mM), KH_2_PO_4_ (0.40 mM), acetic acid (0.1% vol), Hutner’s trace (0.1% vol). Pluronic F-127 powder was dissolved in the medium at 4 °C for 5 hours and under vigorous stirring. Note that in the new Tris-Acetate-Phosphate-Pluronic medium, just like in the traditional Tris-Acetate-Phosphate medium, acetate is used as the carbon source for microalgal growth. Final concentrations of pluronic in TAPP media were selected to be 18%, 20% and 22% (weight percent) in order to obtain a range of T_g_ suitable for the microalgal cultivation and harvesting application.

Cultivation of the wild type microalgae *Chlamydomonas reinhardtii* CC-124, obtained from the Chlamydomonas Resource Center (University of Minnesota, St. Paul, Minnesota), was performed in the TAPP medium as follows: Aliquots of 0.5 ml from liquid subcultures prepared five days preceding the experiment were mixed with 50 ml of TAPP medium at 15 °C (below T_g_ for all the samples). Subsequently, vials containing microalgal culture were placed on a rotary shaker in a room continuously illuminated by full spectrum compact fluorescent lamps (CFL 60 W, Fancierstudio, San Francisco, California) with the photosynthetic active radiation at the top surface of the culture at 100 ± 5 μE m^−2^ s^−1^ and the temperature at 22 ± 1 °C. After seven days of cultivation, the resulting TAPP-microalgae matrix was used for thermorheological characterization and biomass production analyses.

### Rheometry

The rheological experiments to characterize the properties of the TAPP medium were performed using a Combined Motor and Transducer AR-G2 rheometer from TA instruments (New Castle, Delaware). The cone-and-plate geometry with a diameter of 40 mm and a cone angle of 0° 59′ 49″ was used for all the measurements. The temperature control was achieved by a peltier plate using thermoelectric effects to control the temperature accurately and water circulation for rapid heating and cooling over a temperature range of 0 to 100 °C. Strain sweep measurements were performed on the TAPP media samples in order to determine the experimental parameters for the linear viscoelastic (LVE) regime in which the elastic and loss moduli were measured using small amplitude oscillatory shear flow experiments. It was found that for all the samples, a 0.5% strain at a frequency of 1 Hz was appropriate for measuring the LVE properties.

### Characterization of microalgal biomass production and harvesting

The effects of pluronic presence on microalgal cultivation and biomass production were assessed using different analytical techniques. Microalgal biomass production was evaluated through optical density measurements and gravimetric quantification. Microalgal biomass carbohydrate content was assessed using the phenol-sulfuric acid method^35^ and lipids content was assessed through a modified Bligh and Dyer method and fluorescence scanning using Nile Red dye^36^,^37^,^38^,^39^. The impacts of the pluronic-based environment on shape and size of microalgal cells were systematically analyzed using an Axio Imager M1 microscope and a ZEN pro software (Carl Zeiss Inc., Berlin, Germany). These same tools were also used to characterize the distribution of microalgal cells (clusters etc.) in TAPP media with different pluronic concentrations as well as the traditional TAP medium used as control. The shape and size of microalgal clusters were characterized in order to predict the settling velocity for harvesting. The form factor (*FF)* characterizing the deviation from a circle and the equivalent diameter (*De*) of microalgal clusters were computed as presented by Grijspeerdt and Verstraete^29^:


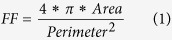



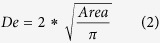


The settling velocity (*V*) of microalgal clusters during the harvesting process could be approximated using Stokes’ law as long as the form factor concurred with a spherical shape and the Reynold number fell within the Stokes’ regime. Under such conditions the settling velocity was calculated as^30^:


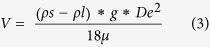


where *ρs* and *ρl* are solid and liquid densities, *g* the acceleration due to gravity, *De* the equivalent diameter and *μ* the solvent viscosity. The average settling rate was then experimentally monitored by allowing microalgae to settle at 15 °C in columns with 10 cm working height and by taking regular optical density measurements (OD_675_) of the broths. To estimate the settling rate, the percent recovery at each measurement time was computed as follows^37^:


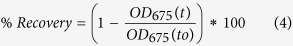


where *OD*_*675*_*(t)* is the optical density of the broth after a settling time (*t*) and *OD*_*675*_*(to)* the optical density at the beginning of the settling experiment or time (*to*).

### Statistical analyses

The microalgal cultivation and harvesting experiments and the related biochemical and rheological analyses mentioned previously were repeated at least 10 times with triplicate measurements for each run. Statistical analyses over the data collected were performed using Minitab software. The results with P-Value less than 0.05 (t-test) were considered statistically significant.

## Additional Information

**How to cite this article:** Estime, B. *et al*. Cultivation and energy efficient harvesting of microalgae using thermoreversible sol-gel transition. *Sci. Rep.* **7**, 40725; doi: 10.1038/srep40725 (2017).

**Publisher's note:** Springer Nature remains neutral with regard to jurisdictional claims in published maps and institutional affiliations.
